# PSA, stage, grade and prostate cancer specific mortality in Asian American patients relative to Caucasians according to the United States Census Bureau race definitions

**DOI:** 10.1007/s00345-020-03242-8

**Published:** 2020-05-26

**Authors:** Marina Deuker, L. Franziska Stolzenbach, Angela Pecoraro, Giuseppe Rosiello, Stefano Luzzago, Zhe Tian, Fred Saad, Felix K.-H. Chun, Pierre I. Karakiewicz

**Affiliations:** 1 Department of Urology, University Hospital Frankfurt, Theodor-Stern Kai 7, 60590 Frankfurt am Main, Germany; 2 Cancer Prognostics and Health Outcomes Unit, Division of Urology, University of Montréal Health Center, Montréal, Québec Canada; 3 Martini-Klinik Prostate Cancer Center, University Hospital Hamburg-Eppendorf, Hamburg, Germany; 4 Department of Urology, San Luigi Gonzaga Hospital, University of Turin, Turin, Italy; 5 Department of Urology and Division of Experimental Oncology, URI, Urological Research Institute, IRCCS San Raffaele Scientific Institute, Milan, Italy; 6 European Institute of Oncology, Milan, Italy

**Keywords:** Prostate cancer survival, Racial disparities, NHOPI, AANHPI, Pacific Islander, Native Hawaiian, AAPI, SEER

## Abstract

**Background:**

The United States Census Bureau recommends distinguishing between “Asians” vs. “Native Hawaiians or Other Pacific Islanders” (NHOPI). We tested for prognostic differences according to this stratification in patients with prostate cancer (PCa) of all stages.

**Methods:**

Descriptive statistics, time-trend analyses, Kaplan–Meier plots and multivariate Cox regression models were used to test for differences at diagnosis, as well as for cancer specific mortality (CSM) according to the Census Bureau’s definition in either non-metastatic or metastatic patients vs. 1:4 propensity score (PS)-matched Caucasian controls, identified within the Surveillance, Epidemiology and End Results database (2004–2016).

**Results:**

Of all 380,705 PCa patients, NHOPI accounted for 1877 (0.5%) vs. 23,343 (6.1%) remaining Asians vs. 93.4% Caucasians. NHOPI invariably harbored worse PCa characteristics at diagnosis. The rates of PSA ≥ 20 ng/ml, Gleason ≥ 8, T3/T4, N1- and M1 stages were highest for NHOPI, followed by Asians, followed by Caucasians (PSA ≥ 20: 18.4 vs. 14.8 vs. 10.2%, Gleason ≥ 8: 24.9 vs. 22.1, vs. 15.9%, T3/T4: 5.5 vs. 4.2 vs. 3.5%, N1: 4.4 vs. 2.8, vs. 2.7%, M1: 8.3 vs. 4.9 vs. 3.9%). Despite the worst PCa characteristics at diagnosis, NHOPI did not exhibit worse CSM than Caucasians. Moreover, despite worse PCa characteristics, Asians exhibited more favorable CSM than Caucasians in comparisons that focussed on non-metastatic and on metastatic patients.

**Conclusions:**

Our observations corroborate the validity of the distinction between NHOPI and Asian patients according to the Census Bureau’s recommendation, since these two groups show differences in PSA, grade and stage characteristics at diagnosis in addition to exhibiting differences in CSM even after PS matching and multivariate adjustment.

## Introduction

According to the official recommendation of the United States (US) Census Bureau, Asian American and Pacific Islander (AAPI) race should be referred to as either being “Native Hawaiian or Other Pacific Islander” (NHOPI) or being “Asian” [1–3]. Despite the presence of this recommendation, no formal study tested its validity, when it is applied to prostate cancer (PCa) patients. Today, Asian Americans account for 4.8% of the total US population with nearly 15 million people [4]. By the year 2060, the Asian community is estimated to have more than doubled compared to an only moderate increase in the total US population [5]. But despite their increasing numerous importance, Asian Americans remain among the most understudied racial minority groups in the US, because Asian race is composed of a variety of heterogeneous groups with a tremendous diversity in socioeconomic status, access to resources, migration patterns, and health characteristics [6]. Specifically, regarding health characteristics, there is a critical need for disaggregation of broad Asian data by ethnic subgroups.

In consequence, we tested for differences in pathological characteristics at diagnosis (prostatic-specific antigen (PSA), grade and stage) as well as for cancer specific mortality (CSM) when the Census Bureau’s definition is applied and comparisons are made with Caucasian patients as a control group. We hypothesized that NHOPI exhibit clinically and statistically meaningful differences in PSA, grade and stage at diagnosis as well as in CSM relative to Asians using Caucasians as control. For purpose of all analyses, we relied on the most contemporary Surveillance, Epidemiology and End Results database (SEER database) between 2004 and 2016.

## Materials and methods

### Study population

The SEER database samples 34.6% of the United States and approximates the United States in terms of demographic composition, as well as of cancer incidence [7]. Within the SEER database (2004–2016), we identified patients ≥ 18 years old (excluded *n* = 1), with known racial/ethnical information not other than Asian or Caucasian (excluded *n* = 106,178), with histologically confirmed PCa diagnosis (International Classification of Disease for Oncology [ICD-O-3] code 8140/3 site code C61.9, excluded *n* = 21,333) and with PSA value (excluded *n* = 89,467). Patients with clinical stage T0 (*n* = 524) or unknown metastatic status (*n* = 13,551), as well as death certificate only and autopsy cases (*n* = 306) were excluded. According to the official Census Bureau of the United States, NHOPI are defined as persons having origins in any of the original peoples of Hawaii, Guam, Samoa, or other Pacific Islands. Conversely, persons having origins in any of the original peoples of the Far East, Southeast Asia, or the Indian subcontinent are defined as Asians [1]. To apply this to the currently used categories in the SEER database, we recoded the Asian or Pacific Islander (AAPI) category according to the reported ethnic subgroups, which resulted in an overall cohort (*n* = 380,705) across all disease stages of 355,485 Caucasian, 23,343 Asians and 1877 NHOPI eligible patients according to the Census Bureau’s recommendation.

Subgroups consisted of patients without lymph node or distant metastases (T_any_N_0_M_0_) (Caucasian *n* = 331,243, Asian *n* = 21,504, NHOPI *n* = 1667) and patients with lymph node and/ or distant metastases (T_any_ N_1_ and/or M_1_, henceforth referred to as T_any_N_1_ and/or M_1_) (Caucasian *n* = 20,035, Asian *n* = 1526, NHOPI *n* = 192).

### Statistical analysis

Descriptive statistics included frequencies and proportions for categorical variables. Means, medians, and interquartile ranges (IR) were reported for continuously coded variables. The Chi-square tested the statistical significance in proportions’ differences. The *t*-test and Kruskal–Wallis test examined the statistical significance of means’ and medians’ differences. Temporal trend analyses were performed to assess annual proportions of PCa characteristics, based on a log-linear regression function.

To maximally reduce the differences between the racial groups, 1:4 propensity score (PS) was performed matching each AAPI category to their Caucasian counterparts for age at diagnosis, PSA, biopsy Gleason Grade Groups [8], clinical stage (cT1, cT2, cT3, cT4, TX), lymph node status (N0, N1, NX), metastatic status (M0, M1) and treatment type (radical prostatectomy [RP], radiotherapy [RT] or no local treatment [NLT]), respectively, within the T_any_N_0_M_0_ and in the T_any_N_1_ and/or M_1_ subgroup.

The endpoint consisted of CSM within Kaplan–Meier Survival plots and multivariate Cox regression analyses. CSM was defined as death attributable to PCa. In all analyses, the predictor of interest consisted of NHOPI vs. Asian vs. Caucasian race. In all statistical analyses, R software environment for statistical computing and graphics (version 3.6.1) was used. All tests were two sided with a level of significance set at p < 0.05.

## Results

### Descriptive characteristics of the study population

Within the SEER database, 380,705 eligible PCa patients across all disease stages were identified. Of all, NHOPI accounted for 1877 (0.5%) vs. 23,343 (6.1%) remaining Asians vs. 355,485 (93.4%) Caucasians. The overall patient and cancer characteristics are displayed in Table 1, stratified according to race: Native Hawaiian or Other Pacific Islander (NHOPI) vs. Asian vs. Caucasian. In general, NHOPI exhibited the worst PSA, grade and stage characteristics, followed by Asians. Conversely, Caucasians showed in general more favorable characteristics for each category. Despite those differences, rates of NLT, RP and RT did not show meaningful differences.

**Table 1:** Descriptive characteristics prostate cancer patients identified within the surveillance, epidemiology and end results database between 2004 and 2016, stratified according to race: Native Hawaiian or Other Pacific Islander (NHOPI) vs. Asian vs. Caucasian

Variables	NHOPI	Asian	Caucasian	Chi-square/Anova test
	1877 (0.50%)	23,343 (6.1%)	355,485 (93.40%)	*p*-value
Age at diagnosis*, *years				
Median (interquartile range)	66 (61–72)	67 (62–74)	66 (60–72)	< 0.001
PSA, ng/ml				
Median (interquartile range)	7.9 (5.5–14.7)	7.6 (5.3–12.6)	6.3 (4.6–9.8)	< 0.001
Gleason grade groups*, n *(%)				
I (Gleason score 6)	616 (32.8)	8221 (35.2)	142,363 (40)	< 0.001
II (Gleason score 3 + 4)	438 (23.3)	5613 (24)	93,261 (26.2)	
III (Gleason score 4 + 3)	259 (13.8)	2960 (12.7)	39,867 (11.2)	
IV (Gleason score 8)	229 (12.2)	2774 (11.9)	29,901 (8.4)	
V (Gleason scores 9–10)	239 (12.7)	2391 (10.2)	26,587 (7.5)	
Unknown	96 (5.1)	1384 (5.9)	23,506 (6.6)	
Clinical T stage*, n *(%)				
cT1	1069 (57)	14,602 (62.6)	215,131 (60.5)	< 0.001
cT2	609 (32.4)	6717 (28.8)	115,068 (32.4)	
cT3	72 (3.8)	734 (3.1)	9716 (2.7)	
cT4	31 (1.7)	258 (1.1)	2900 (0.8)	
cTX	96 (5.1)	1032 (4.4)	12,670 (3.6)	
N stage*, n *(%)				
N0	1740 (92.7)	22,107 (94.7)	338,599 (95.2)	< 0.001
N1	83 (4.4)	663 (2.8)	9736 (2.7)	
NX	54 (2.9)	573 (2.5)	7150 (2)	
M stage*, n *(%)				
M0	1720 (91.6)	22,206 (95.1)	341,496 (96.1)	< 0.001
M1	157 (8.4)	1137 (4.9)	13,989 (3.9)	
Treatment*, n *(%)				
No local treatment	494 (26.3)	6123 (26.2)	86,916 (24.4)	< 0.001
Radical prostatectomy	652 (34.7)	8423 (36.1)	138,334 (38.9)	
Radiotherapy	682 (36.3)	8335 (35.7)	122,033 (34.3)	
Unknown treatment	49 (2.6)	462 (2)	8202 (2.3)	

**Fig. 1:**
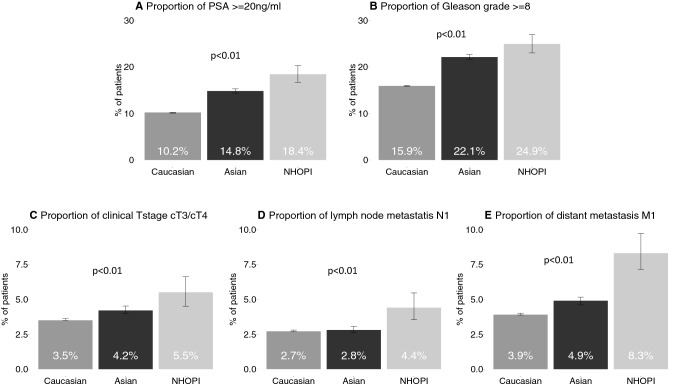
Barplots depicting the proportion of Native Hawaiian or Other Pacific Islander (NHOPI) vs. Asian vs. Caucasian patients with PSA≥20ng/ml (**a**), Gleason grade ≥8 (**b**), clinical stages cT3–cT4 (**c**), lymph node metastasis (**d**) and distant metastasis (**e**).

As graphically depicted in Fig. 1, NHOPI harbored worse PCa characteristics at diagnosis, than Asians and Asians harbored worse PCa characteristics at diagnosis than Caucasians. NHOPI had highest rates of high PSA relative to Asians and Caucasians (PSA ≥ 20 ng/ml: 18.4 [NHOPI] vs. 14.8 [Asian] vs. 10.2% [Caucasian]), and highest rates of high-grade Gleason score (Gleason ≥ 8: 24.9 vs. 22.1, vs. 15.9%). Similarly, the rates of stage T3/T4 (5.5 vs. 4.2 vs. 3.5%), N1 (4.4 vs. 2.8, vs. 2.7%) and M1 (8.3 vs. 4.9 vs. 3.9%) were also highest in NHOPI relative to Asians and Caucasians (Fig. 1a–e).

### Annual rates of PSA, grade and stage proportions according to race

**Fig. 2:**
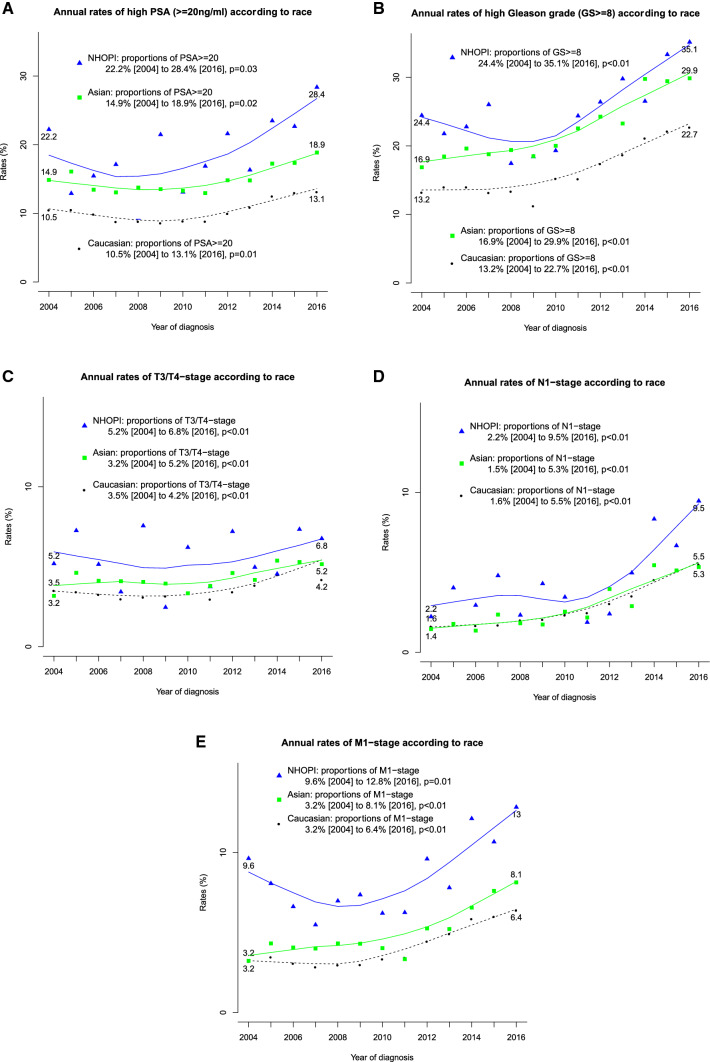
Annual rates of proportions of high PSA (**a**), high Gleason grade (**b**), T3/4 stage (**c**) N1 stage (**d**) and M1 stage (**e**) stratified according to race: Native Hawaiian or Other Pacific Islander (NHOPI) vs. Asian vs. Caucasian patients

In three out of five annual trend analyses, NHOPI exhibited highest rates of, respectively, PSA greater than 20 ng/ml, Gleason greater than 8 and M1 stage. Conversely, in the two remaining time trend analyses, T3/T4 stage and N1 stage, annual proportions indicated a tendency towards higher rates in NHOPI followed by Asians and followed by Caucasians in that order, at least in the most contemporary years (Fig. 2a–e).

### Propensity score (PS) matching, 1:4

To adjust for important differences in PSA, grade and clinical stage, we relied on 1:4 PS matching for the purpose of four specific comparisons: First, we tested for CSM differences between NHOPI vs. Caucasians in the non-metastatic subgroup. Second, we tested for CSM differences between NHOPI vs. Caucasians in the metastatic subgroup. Third, we tested for CSM differences between Asians vs. Caucasians in the non-metastatic subgroup. Fourth, we tested for CSM differences between Asians vs. Caucasians in the metastatic subgroup. In all comparisons, matching of NHOPI or Asian cases with Caucasian controls resulted in a standard mean difference after matching of less than 0.1 for all variables included in matching. After PS matching, no residual statistically significant or clinically meaningful differences remained between non-metastatic and metastatic NHOPI and Caucasians (Table 2A and B), Similarly, after PS matching between metastatic Asians and metastatic Caucasians, no residual statistically significant differences remained (Table 2D); whereas between non-metastatic Asians and non-metastatic Caucasians, still marginal statistically significant differences remained (Table 2C) which are of no clinical relevance.

**Table 2:** Population of non-metastatic NHOPI and Caucasian patients after 1:4 PS matching

Panel A				
Variable	Cat/Stat	NHOPI	Caucasians	*p* t/chi
		*N* = 1 667	*N* = 6 668	
Age	Mean	66.1 (0.198)	66	0.62
	(STE)		(0.103)	
PSA	Mean	12.3 (0.377)	11.8	0.31
	(STE)		(0.198)	
Clinical T stage	cT1	1018 (61.1)	4276 (64.1)	0.11
	cT2	546 (32.8)	2047 (30.7)	
	cT3	49 (2.9)	182 (2.7)	
	cT4	5 (0.3)	20 (0.3)	
	cTX	49 (2.9)	143 (2.1)	
GS diagnoses	I	604 (36.2)	2334 (35)	0.96
	II	429 (25.7)	1764 (26.5)	
	III	241 (14.5)	975 (14.6)	
	IV	183 (11)	730 (10.9)	
	V	156 (9.4)	644 (9.7)	
	Unknown	54 (3.2)	221 (3.3)	
Treatment	NLT	362 (21.7)	1421 (21.3)	0.63
	RP	626 (37.6)	2454 (36.8)	
	RT	643 (38.6)	2670 (40)	
	Unknown	36 (2.2)	123 (1.8)	

Panel B				
Variable	Cat/Stat	NHOPI	Caucasians	*p* t/chi
		*N* = 192	*N* = 768	
Age	Median (Range)	66	67	0.43
		(61.8–73)	(61–74.2)	
PSA	Median (Range)	85.8	98	0.15
		(18.1–98)	(25.9–98)	
Clinical T stage	cT1	43 (22.4)	175 (22.8)	0.92
	cT2	57 (29.7)	230 (29.9)	
	cT3	22 (11.5)	81 (10.5)	
	cT4	26 (13.5)	89 (11.6)	
	cTX	44 (22.9)	193 (25.1)	
GS diagnoses	I	7 (3.6)	17 (2.2)	0.36
	II	6 (3.1)	28 (3.6)	
	III	17 (8.9)	72 (9.4)	
	IV	43 (22.4)	126 (16.4)	
	V	79 (41.1)	350 (45.6)	
	Unknown	40 (20.8)	175 (22.8)	
Treatment	NLT	122 (63.5)	504 (65.6)	0.66
	RP	25 (13)	79 (10.3)	
	RT	34 (17.7)	148 (19.3)	
	Unknown	11 (5.7)	37 (4.8)	
Metastatic status	N1	35 (18.2)	108 (14.1)	0.18
	M1	157 (81.8)	660 (85.9)	

Panel C				
Variable	Cat/Stat	Asian	Caucasians	p t/chi
		*N* = 21 504	*N* = 86 016	
Age	Median (Range)	67	67	0.62
		(62–73)	(62–73)	
PSA	Median (Range)	7.2	6.7	< 0.01
		(5.2–11.3)	(5.0–10.0)	
Clinical T stage	cT1	14,017 (65.2)	57,317 (66.6)	< 0.01
	cT2	6186 (28.8)	23,549 (27.4)	
	cT3	520 (2.4)	1747 (2)	
	cT4	75 (0.3)	247 (0.3)	
	cTX	706 (3.3)	3156 (3.7)	
GS diagnoses	I	8057 (37.5)	33,109 (38.5)	< 0.01
	II	5461 (25.4)	21,483 (25)	
	III	2779 (12.9)	11,364 (13.2)	
	IV	2398 (11.2)	9614 (11.2)	
	V	1693 (7.9)	6453 (7.5)	
	Unknown	1116 (5.2)	3993 (4.6)	
Treatment	NLT	5116 (23.8)	20,098 (23.4)	< 0.01
	RP	8080 (37.6)	33,423 (38.9)	
	RT	7889 (36.7)	31,099 (36.2)	
	Unknown	419 (1.9)	1396 (1.6)	

Panel D				
Variable	Cat/Stat	Asians	Caucasians	*p* t/chi
		*N* = 1 526	*N* = 6 104	
Age	Median (Range)	69	69	0.84
		(63–77)	(62–77)	
PSA	Median (Range)	57.2	66.7	0.24
		(15.7–98)	(15.7–98)	
Clinical T stage	cT1	414 (27.1)	1683 (27.6)	0.95
	cT2	446 (29.2)	1818 (29.8)	
	cT3	200 (13.1)	805 (13.2)	
	cT4	173 (11.3)	659 (10.8)	
	cTX	293 (19.2)	1139 (18.7)	
GS diagnoses	I	41 (2.7)	141 (2.3)	0.76
	II	109 (7.1)	409 (6.7)	
	III	138 (9)	599 (9.8)	
	IV	332 (21.8)	1268 (20.8)	
	V	662 (43.4)	2705 (44.3)	
	Unknown	244 (16)	982 (16.1)	
Treatment	NLT	874 (57.3)	3644 (59.7)	0.27
	RP	303 (19.9)	1181 (19.3)	
	RT	317 (20.8)	1146 (18.8)	
	Unknown	32 (2.1)	133 (2.2)	
Metastatic status	N1	389 (25.5)	1531 (25.1)	0.77
	M1	1137 (74.5)	4573 (74.9)	

### PS-matched Kaplan–Meier (KM) and PS-matched multivariate Cox regression models

#### Non-metastatic (T_any_N_0_M_0_) NHOPI vs. Caucasian patients

**Fig. 3:**
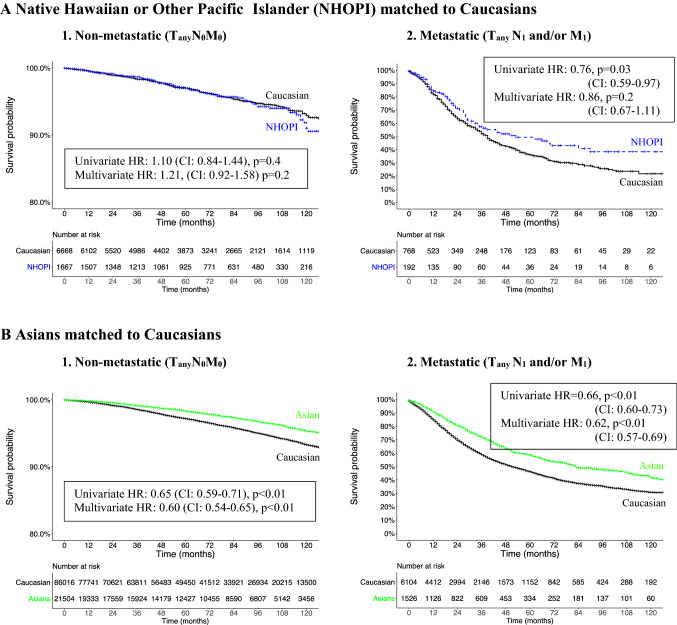
Propensity score (PS)-matched Kaplan–Meier plots accompanied by PS-matched uni- and multivariably adjusted hazard ratios (HR) predicting CSM in **a** NHOPI and **b** Asians relative to Caucasians. Comparisons are stratified according to non-metastatic (T_any_N_0_M_0_) and metastatic (T_any_N_1_and/or M_1_) disease stages. The first comparison focuses on NHOPI vs. Caucasians, the second comparison on Asian vs. Caucasian prostate cancer patients within stratifications according to non-metastatic (1) vs. metastatic (2) stage after 1:4 propensity score matching for age at diagnosis, PSA, Gleason grade, clinical stage and treatment type. Multivariate adjustment was made for age at diagnosis, PSA, Gleason grade, clinical stage and treatment type

After PS matching between NHOPI and Caucasians in non-metastatic (T_any_N_0_M_0_) patients, 10-year CSM-free rates were 91.0 in NHOPI vs. 93.3% in Caucasians (*p* = 0.4). In multivariate Cox regression analyses predicting CSM after adjustment for stage at presentation, NHOPI race did not reach independent predictor status (HR = 1.21, *p* = 0.2) for CSM relative to Caucasians (Fig. 3a1).

#### Metastatic (T_any_N_1_ and/or M_1_) NHOPI vs. Caucasian patients

After PS matching between NHOPI and Caucasians in metastatic (T_any_N_1_ and/or M_1_) patients, 10-year CSM-free rates were 38.3% in NHOPI vs. 21.2% in Caucasians (*p* = 0.03). In multivariate Cox regression analyses predicting CSM after adjustment for stage at presentation, NHOPI race did not reach independent predictor status (HR = 0.86, *p* = 0.2) relative to Caucasians (Fig. 3a2).

### Non-metastatic (T_any_N_0_M_0_) Asian vs. Caucasian patients

After PS matching between Asians and Caucasians in non-metastatic (T_any_N_0_M_0_) patients, 10-year CSM-free rates were 95.3 in Asians vs. 93.2% in Caucasians (*p* < 0.01). In multivariate Cox regression analyses predicting CSM after adjustment for stage at presentation, Asian race remained a significant predictor (HR 0.60, *p* < 0.01) for favorable CSM relative to Caucasians (Fig. 3b1),

### Metastatic (T_any_N_1_ and/or M_1_) Asian vs. Caucasian patients

After PS matching between Asians and Caucasians in metastatic (T_any_N_1_ and/or M_1_) patients, 10-year CSM-free rates were 41.9 in Asians vs. 30.6% in Caucasians (*p* < 0.01). In multivariate Cox regression analyses predicting CSM after adjustment for stage at presentation, Asian race remained a significant predictor (HR 0.62, *p *< 0.01) for favorable CSM relative to Caucasians (Fig. 3b2).

## Discussion

The objective of this study was to validate of the distinction between NHOPI and Asian patients regarding PCa stage at presentation and survival after treatment according to the Census Bureau’s recommendation. We hypothesized that NHOPI exhibit clinically and statistically meaningful differences in PCa stage and grade at presentation, as well as in CSM after diagnosis relative to Asians. Our results provided several important observations:

First, we documented important differences in PSA, grade and stage at diagnosis. Specifically, NHOPI harbored substantially worse PCa characteristics at diagnosis, than their Asian or Caucasian counterparts evidenced by highest rates of PSA over 20 ng/ml (18.4 [NHOPI] vs. 14.8 [Asian] vs. 10.2% [Caucasian]), highest rates of Gleason score ≥ 8 (24.9 vs. 22.1, vs. 15.9%), highest rates of stage-T3/T4 at diagnosis (5.5 vs. 4.2 vs. 3.5%), highest rates of stage N1 at diagnosis (4.4 vs. 2.8, vs. 2.7%) and highest rates of stage M1 at diagnosis (8.3 vs. 4.9 vs. 3.9%). To the best of our knowledge, we are the first to apply the Census Bureau’s definition of NHOPI and Asians within PCa patients. The recorded differences between these two groups validate the pertinence of the Census Bureau’s definition in the context of PSA, grade and stage at diagnosis. Previous investigators have not used the Census Bureau recommended definition, but instead relied on other stratifications of AAPI patients. With one exception [9], AAPI patients generally harbored less favorable PSA, grade and stage characteristics than their Caucasian counterparts [10–13].

Second, we examined the annual proportions of patients with high PSA, high Gleason grade, T3/T4 stage, N1 or M1 stage within NHOPI, Asian and Caucasian race over time. Our analyses showed highest rates of unfavorable tumor characteristics in NHOPI that were followed in absolute rates by Asians and Caucasians, in that order. To the best of our knowledge, we are the first to perform annual rates’ analyses of PCa characteristics at diagnosis according to Census Bureau’s definitions of NHOPI vs. Asian race groups. In consequence, we cannot compare these results with any other study. Nonetheless, it should be noted that the rates of unfavorable grade and stage distribution have also risen over time in Caucasian patients, at a relatively similar pace to their NHOPI and Asian counterparts. This finding is in agreement with two recently published studies that also found rising incidence of primary metastatic PCa in population-based analyses [14, 15].

Third, to account for important differences in PSA, grade and stage between NHOPI, Asians, and Caucasians, we relied on 1:4 PS matching to compare CSM between these racial groups. First, we found no statistically significant CSM differences between NHOPI and Caucasians in either non-metastatic or metastatic PCa stage. Second, we found more favorable CSM in Asians, relative to Caucasians in both non-metastatic and metastatic PCa stage (multivariate HR 0.59 in T_any_N_0_M_0_ patients and 0.62 in T_any_N_1_ and/or M_1_ patients). These findings suggest that after accounting for important PSA, grade and stage differences at diagnosis, NHOPI race does not exert a prognostic effect on CSM. Conversely, the presence of Asian race exerts a prognostically favorable effect on CSM, relative to Caucasians. These observations are in accordance with several reports that focused on Asian Americans as a whole, without applying the Census Bureaus’ definition of either NHOPI or other Asian race [10–13]. The stage and grade differences at presentation between NHOPI, Asians and Caucasians may be attributed to differences in congenital and/or acquired risk factors for PCa that were previously suggested by several investigators [16, 17]. However, to the best of our knowledge, there are no data explaining why differences in stage at presentation do not translate into prognostically worse outcomes after treatment in both NHOPI, as well as Asians.

It is of note that a more historical study by Goggins et al. [18], which also relied on the SEER database (1991–2004), made similar observations. Contrary to our work, they assessed various tumor sites and did not apply the Census Bureau recommendation. However, in analyses according to ethnicity, the authors found that Samoans were most likely to present with advanced disease and had the worst CSM for all sites considered. Our work distinguishes itself from this, as well from other previous reports [10, 13, 16, 19], where CSM was examined without PS matching and without multivariate analyses. In those reports, NHOPI and its ethnic subgroups exhibited higher CSM than other races, due to lack of adjustment for unfavorable stage at diagnosis. This hypothesis is also supported by a work by Islam et al. [6] stating that much of the survival disadvantage recorded for Pacific Islanders originates from late diagnosis. Thus, it is possible that the presence of more advanced stage in NHOPI, as well as in Asians, is related to fewer screening or diagnostic opportunities, in addition to potential treatment delays relative to their Caucasian counterparts. This explanation is also supported by the results of a recent work by Falagario et al. [20] on racial disparities, which reported no difference between African Americans and Caucasians in pathologic outcomes after RP. Their findings suggest that access to and use of advanced diagnostic tests may help mitigate PCa racial disparities.

Regardless of its cause, efforts should ideally eliminate the presence of PSA, stage and grade differences at diagnosis that distinguish NHOPI and Asians from their Caucasian counterparts. We are not the first to make such recommendations, Chao et al., Trinh et al. and Robbins et al. [10, 13, 16] previously made similar recommendations. Unfortunately, our findings indicate that they have not been implemented into clinical practice and have not resulted in the reduction of unfavorable PSA, grade and stage characteristics in either NHOPI or Asian relative to Caucasians over time. In consequence, further or renewed efforts are needed.

Our work has limitations and should be interpreted in the context of its retrospective and population based design. It should be noted, that the numbers of NHOPI patients were relatively small. However, only the NCBD (National Cancer DataBase) may provide a similar perspective regarding AAPI patients. In consequence, even a more limited patient cohort is of great value and the worth of its contribution should not be underestimated. Furthermore, despite best efforts aimed at PS matching, retrospective analyses and PS matching for known and available variables may still suffer from remaining differences related to unmeasured or unavailable confounding variables. As in all SEER-based analyses, comorbidities were not available and could lead to residual confounding effects in CSM analyses. Finally, the SEER database only includes North American patients and our findings are only applicable to Asians from the United States and may not be generalizable to Asians from other parts of the world. These, as well as all other limitations related to the retrospective, population-based nature of the SEER database, apply to this, as well as to other similar analyses that were based on the SEER database or other similar large-scale data repositories, such as NCDB, NIS (National Inpatient Sample) or NSQIP (National Surgical Quality Improvement Program).

## Conclusion

Our observations corroborate the validity of the distinction between NHOPI and Asian patients according to the Census Bureau’s recommendation, since these two groups show differences in PSA, grade and stage characteristics at diagnosis in addition to exhibiting differences in CSM even after PS matching and multivariate adjustment. Specifically, NHOPI and Asians exhibit more unfavorable stage and grade at presentation than Caucasians. Moreover, Asians exhibit a CSM advantage; whereas, NHOPI exhibit no differences in CSM after PS matching and multivariate adjustment. In consequence, given the different cancer profiles, our results show that there is a need for disaggregation of AAPI data according to the official recommendation of the United States Census Bureau.
